# RNA‐sequencing reveals differential fibroblast responses to bleomycin and pneumonectomy

**DOI:** 10.14814/phy2.16148

**Published:** 2024-07-11

**Authors:** Jack H. Wellmerling, Sara R. Dresler, Jeffrey A. Meridew, Kyoung M. Choi, Daniel J. Tschumperlin, Qi Tan

**Affiliations:** ^1^ Department of Physiology and Biomedical Engineering Mayo Clinic College of Medicine and Science Rochester Minnesota USA; ^2^ The Hormel Institute, University of Minnesota Austin Minnesota USA

**Keywords:** bleomycin, ITPKC, lung fibroblasts, pneumonectomy, pulmonary fibrosis, RNA‐sequencing

## Abstract

Pulmonary fibrosis is characterized by pathological accumulation of scar tissue in the lung parenchyma. Many of the processes that are implicated in fibrosis, including increased extracellular matrix synthesis, also occur following pneumonectomy (PNX), but PNX instead results in regenerative compensatory growth of the lung. As fibroblasts are the major cell type responsible for extracellular matrix production, we hypothesized that comparing fibroblast responses to PNX and bleomycin (BLM) would unveil key differences in the role they play during regenerative versus fibrotic lung responses. RNA‐sequencing was performed on flow‐sorted fibroblasts freshly isolated from mouse lungs 14 days after BLM, PNX, or sham controls. RNA‐sequencing analysis revealed highly similar biological processes to be involved in fibroblast responses to both BLM and PNX, including TGF‐β1 and TNF‐α. Interestingly, we observed smaller changes in gene expression after PNX than BLM at Day 14, suggesting that the fibroblast response to PNX may be muted by expression of transcripts that moderate pro‐fibrotic pathways. *Itpkc*, encoding inositol triphosphate kinase C, was a gene uniquely up‐regulated by PNX and not BLM. ITPKC overexpression in lung fibroblasts antagonized the pro‐fibrotic effect of TGF‐β1. RNA‐sequencing analysis has identified considerable overlap in transcriptional changes between fibroblasts following PNX and those overexpressing ITPKC.

## 1 INTRODUCTION

Idiopathic pulmonary fibrosis (IPF) is an incurable interstitial lung disease of unknown origin with a median survival of 2–3 years following diagnosis (Ley et al., 2011). IPF is characterized by excessive deposition of collagenous scar tissue that does not resolve. The strongest risk factor for developing IPF is age, but tobacco smoking, exposure to environmental dusts, microbial agents, and genetics also contribute (Raghu et al., 2011). The pathogenesis of IPF remains incompletely understood. Many cellular compartments have been suggested to contribute to IPF, including epithelial, mesenchymal, and immune cells. Fibroblasts are the cell type primarily responsible for collagen production in IPF (Tsukui et al., 2020). The progressive, nonresolving nature of IPF necessitates a deeper understanding of fibroblast biology and identification of differential responses of fibroblasts during regulated regenerative processes and dysregulated fibrotic responses.

While animal models of lung fibrosis and repair are imperfect, they offer the potential to compare cellular responses at carefully timed intervals after the initiation of fibrotic versus regenerative responses. Intratracheal bleomycin (BLM) instillation is a well‐established experimental model of pulmonary fibrosis in mice (Liu et al., 2017). The first three days following bleomycin instillation involve a robust but transient inflammatory response, which is followed by a peak fibroproliferative response that occurs approximately 14 days following administration (Liu et al., 2017). At this time point expression of inflammatory cytokines tend toward to baseline levels, while peak profibrotic gene expression and matrix deposition are observed (Chaudhary et al., 2006; Izbicki et al., 2002; Schiller et al., 2015). Pneumonectomy (PNX) is a surgical procedure in which one lung is removed. Following PNX in small animals, the remaining lung rapidly grows to compensate for lost respiratory surface area (Brody et al., 1978), making it a useful model of a coordinated and regenerative lung repair response. Compensatory lung growth has been documented in a human patient over the course of 15 years (Butler et al., 2012). Post‐PNX lung growth is an orchestrated process involving many cell types, including epithelial proliferation (Fehrenbach et al., 2008), neovascularization (Sakurai et al., 2007), and a type 2 immune response (Lechner et al., 2017). Following PNX in mice, monocyte and macrophage numbers increase noticeably and peak around 7 days post‐surgery (Lechner et al., 2017), and the mass of the remaining lung increases by nearly 50% 14 days post‐PNX (Lechner et al., 2017), a time course similar to the fibrotic response observed post‐BLM. Like BLM responses, myofibroblast contraction is important for realveolarization following PNX (Li et al., 2020), and increased collagen production and fibroblast proliferation have also been implicated in compensatory lung growth (McAnulty et al., 1992). Importantly, PNX‐associated lung regeneration does not result in fibrosis. Contrasting fibroblast responses to these two models thus offers an opportunity to identify shared and unique gene programs involved in fibrotic versus regenerative lung repair.

Previously our group has compared lung fibroblast transcriptomes during fibrosis and resolution following bleomycin in mice (Tan et al., 2021). Here, we hypothesized that investigating how pulmonary fibroblasts differentially respond to PNX compared to BLM could identify biological pathways that are important for ensuring fibrosis does not occur during lung repair and regeneration associated with PNX, and potentially unveil novel therapeutic targets for pulmonary fibrosis. To do this, we employed bulk RNA‐sequencing of lung fibroblasts isolated from mice 14 days after BLM injury, PNX, or sham controls. Our results revealed highly similar biological processes to be involved in fibroblast responses to both BLM and PNX, but with generally smaller changes in gene expression after PNX than BLM, suggesting that the fibroblast response to PNX may be muted by expression of transcripts that moderate pro‐fibrotic pathways. We identified Itpkc, encoding inositol triphosphate kinase C, as a gene uniquely up‐regulated after PNX but not BLM. We tested ITPKC overexpression in human lung fibroblasts where it antagonized the pro‐fibrotic effects of TGF‐β1. RNA‐sequencing analysis of primary human lung fibroblasts overexpressing ITPKC further supported its role in repressing collagen production. Our results thus provide a resource for comparing fibroblast responses during fibrotic versus regenerative responses in the lungs and point toward ITPKC as a potential moderator that attenuates pro‐fibrotic responses during regenerative lung repair.

## 2 METHODS

### 2.1 Mouse experiments

All mouse experiments were performed under a protocol approved by the Mayo Clinic Institutional Animal Care and Use Committee. Col1α1‐GFP reporter mice were obtained as a kind gift from Dr. Derek Radisky and have been described in more detail previously (Yata et al., 2003). Mice were anesthetized with intraperitoneal injection of 90 mg/kg ketamine and 10 mg/kg xylazine prior to procedure. 1 U/kg bleomycin (APP Pharmaceutical) or phosphate buffered saline (PBS, sham) was administered intratracheally using a MicroSprayer (Penn‐Century). Mice were euthanized 14 days after bleomycin administration using intraperitoneal overdose of ketamine and xylazine, and death was confirmed by organ removal.

Pneumonectomy was performed according to published protocols (Liu et al., 2014). Briefly, mice were anesthetized with intraperitoneal injection of 90 mg/kg ketamine and 10 mg/kg xylazine, a surgical window was cut, and the left lung was removed. Following lung removal, a suture clip was used to close the remaining lung, and the surgical window was closed using surgical glue. Mice were euthanized 14 days after pneumonectomy using intraperitoneal overdose of ketamine and xylazine, and death was confirmed by organ removal.

### 2.2 Tissue harvest and flow sorting

Lungs were washed with ice‐cold PBS, and immediately subject to mechanical digestion in a solution of DNAse I (Sigma‐Aldrich, # 4536282001) and Liberase (Sigma‐Aldrich, # 5401127001) in Dulbecco's Modified Eagle's Medium (DMEM, Thermo Fisher Scientific). The resulting tissue was digested for 35 min at 37°C. Following digestion, the digestion enzymes were inactivated with 10% fetal bovine serum (FBS, Thermo Fisher Scientific). Solutions were filtered through a 40 μm filter followed by lysis of red blood cells. Fibroblasts were isolated using a BD FACSMelody flow sorter. Briefly, dead cells were gated out via detection of 4′,6‐diamidino‐2‐phenylindole (DAPI, Thermo Fisher Scientific, #62247). Antibodies were used to detect CD45 (BioLegend, #103132) for leukocytes, epithelial cell adhesion molecule/CD326 (BioLegend, #118214) for epithelial cells, and CD31 (BioLegend, #102408) for endothelial cells. Cells positive for these markers were gated out, and cells positive for GFP of this lineage‐negative population were taken to be fibroblasts. Immediately after sorting, isolated cells were lysed in RNA lysis buffer (QIAGEN, #74004) with 1% β‐mercaptoethanol (Bio‐Rad) and stored at −80°C.

### 2.3 Hydroxyproline assay

Lung hydroxyproline content was measured using a hydroxyproline assay kit (abcam, #ab222941) as previously described (Haak et al., 2019). Briefly, tissue was homogenized in sterile water at a ratio of 10 mg tissue to 100 μL water and hydrolyzed in 12 N HCl at 120°C for 3 h. Hydrolyzed samples were then filtered through a 45 μm Spin‐X® filter (Corning). 10 μL of sample was then dried in a speed vacuum for 2 h, then incubated with 100 μL chloramine T reagent for 5 min at room temperature and 100 μL 4‐(Dimethylamino) benzaldehyde (DMAB) for 90 min at 60°C. The absorbance of oxidized hydroxyproline was determined at 560 nm. Hydroxyproline concentrations were calculated from a standard curve generated using known concentrations of trans‐4‐hydroxyl‐L‐proline. The total amount of protein isolated from the weighed tissues was determined by using a protein assay kit (Bio‐Rad, Hercules, CA, USA) measuring absorbance at 595 nm. The amount of collagen was expressed in μg/mg total protein.

### 2.4 Histology and immunostaining

Mouse lung tissues were inflated and fixed with 4% formaldehyde, then embedded into frozen blocks using Tissue‐Tek OCT compound (Sakura). Ten micrometre frozen sections were used for H&E staining and immunostaining. The H&E staining was performed according to the manufacturer's protocol (Abcam). For immunostaining, tissues were fixed in 4% paraformaldehyde for 10 min and permeabilized with 0.25% Triton X‐100 for 15 min. After blocking with 1% BSA for 1 h, samples were incubated overnight at 4°C with an ITPKC antibody (1:100, Biorbyt, orb184462) in PBS with 1% BSA. This was followed by incubation with a Alexa Fluor 647‐conjugated goat anti‐rabbit secondary antibody (1:500, Thermo Fisher Scientific, A21245) and DAPI (1:1000, Thermo Fisher Scientific) at room temperature for 1 h. All images were captured using a Zeiss LSM 780 confocal microscope or an Olympus CKX53 microscope.

### 2.5 RNA sequencing and analysis

RNA from sorted fibroblasts was isolated using a QIAGEN RNEasy MicroKit (QIAGEN, #74004) according to the manufacturer's instructions. RNA sequencing was performed on sorted fibroblasts from sham, bleomycin‐treated, and pneumonectomized mice using Mayo Clinic's Genome Analysis Core. RNA libraries were prepared using the Illumina cBot and HiSeq 3000 PE Cluster Kit, yielding 33–40 million reads per sample. RNA‐sequencing on cultured normal human lung fibroblasts was conducted by the Beijing Genome Institute (Shenzhen, China). Differential expression analysis was performed by a trained biostatistician and differential expression was determined using the DESeq2 algorithm (Love et al., 2014). Genes were considered differentially expressed if they had a Benjamini‐Hochberg‐adjusted *p*‐value less than 0.05 and an absolute log‐2 fold‐change greater than 1. Venn diagrams were prepared using the webtool Venny (https://bioinfogp.cnb.csic.es/tools/venny/index.html). Heatmaps were prepared using Morpheus (https://software.broadinstitute.org/morpheus). All the RNA‐seq data have been deposited into the GEO databases (GSE161322 and GSE220254).

### 2.6 Pathway enrichment analysis

Pathway enrichment analysis was conducted using Qiagen's Ingenuity Pathway Analysis (IPA) software. Genes differentially expressed in either direction were used for this analysis, which accounts for the magnitude of the fold‐change of individual genes. Upstream regulators were predicted in IPA software and filtered to display only biologic regulators (i.e., genes, mRNAs, and proteins). Kyoto Encyclopedia of Genes and Genomes (KEGG) pathway and gene ontology (GO) enrichment analysis were conducted using the Database for Annotation, Visualization and Integrated Discovery (DAVID) (Huang da et al., 2009).

### 2.7 Motif enrichment prediction

Motif enrichment prediction was performed on RNA‐sequencing data using iRegulon (Janky et al., 2014), an add‐on for the software Cytoscape (Shannon et al., 2003). Enriched motifs associated with either bleomycin, pneumonectomy, or ITPKC overexpression were predicted using significantly up‐regulated genes compared to sham or control.

### 2.8 Cell culture and quantitative real‐time PCR

Primary normal human lung fibroblasts (NHLFs) were kindly provided by Peter Bitterman and Craig Henke at the University of Minnesota under a protocol approved by the University of Minnesota Institutional Review Board. Cells were cultured in DMEM with 10% FBS and a 1% antimicrobial/antifungal solution. Transient transfection was performed using a Lipofectamine 3000 kit (Thermo Fisher Scientific, #L3000008) according to the manufacturer's instructions. ITPKC was overexpressed using plasmid #134603 from Addgene (Dewaste et al., 2003). Quantitative real‐time PCR (qRT‐PCR) was performed by isolating RNA from NHLFs and synthesizing complementary DNA using a SuperScript III cDNA synthesis kit (Thermo Fisher Scientific, #11754050). Recombinant human TGF‐β1 (R&D Systems, #240‐B‐002/CF) was added at a final concentration of 10 ng/mL. 2‐APB (Tocris, #1224) was added at a concentration of 50 μM. Thapsigargin (Tocris, #1138) was added at a concentration of 5 μM. The PCR primers are available in Table 1.

**TABLE 1:** Primers were purchased from Integrated DNA Technologies.

Human GAPDH‐F	AATGAAGGGGTCATTGATGG
Human GAPDH‐R	AAGGTGAAGGTCGGAGTCAA
Human COL1A1‐F	GAGGGCCAAGACGAAGACATC
Human COL1A1‐R	CAGATCACGTCATCGCACAAC
Human ACTA2‐F	GTGTTGCCCCTGAAGAGCAT
Human ACTA2‐R	GCTGGGACATTGAAAGTCTCA
Human CTGF‐F	CAGCATGGACGTTCGTCTG
Human CTGF‐R	AACCACGGTTTGGTCCTTGG
Human FN1‐F	AGGAAGCCGAGGTTTTAACTG
Human FN1‐R	AGGACGCTCATAAGTGTCACC
Human ITPKC‐F	TGGACACAACCTAGCACTGAC
Human ITPKC‐R	TGGTTCCTCTAATGGGCCATC

*Note*: “F” indicates forward and “R” indicates reverse primer sequence.

### 2.9 Western blotting

Cells were lysed in RIPA buffer with Halt protease and phosphatase inhibitor cocktail (Thermo Fisher Scientific). Lysate protein concentration was measure using Pierce BCA protein assay kit (Thermo Fisher Scientific) and 10 μg of protein was run on 4%–15% Mini‐PROTEAN® TGX™ gels (Bio‐Rad). Proteins were transferred to PVDF membranes with Trans‐Blot Turbo transfer system (Bio‐Rad). PVDF membranes were then blocked for 1 h at room temperature with 5% w/v nonfat dry milk in TBST. Membranes were probed with antibodies against COL1A1 (Novus Biologicals, #NB600‐408), α‐SMA (Sigma‐Aldrich, #F3777), and GAPDH (Cell Signaling Technology, #2118) overnight at 4°C. Membranes were then incubated with HRP‐conjugated secondary antibodies (Promega). Bands were detected using Super Signal West Pico Plus (Thermo Fisher Scientific) and imaged using a Bio‐Rad ChemiDoc system.

### 2.10 Statistical analysis

Statistical analysis was performed with Graphpad Prism 9 software. For comparison among multiple groups, one way ANOVA with Fisher's least significant difference as a posthoc test was used. A *p*‐value less than 0.05 was considered significant. All error bars on graphs indicate mean ± standard deviation.

## 3 RESULTS

### 3.1 Pneumonectomy increases collagen production without inducing fibrosis

**FIGURE 1:**
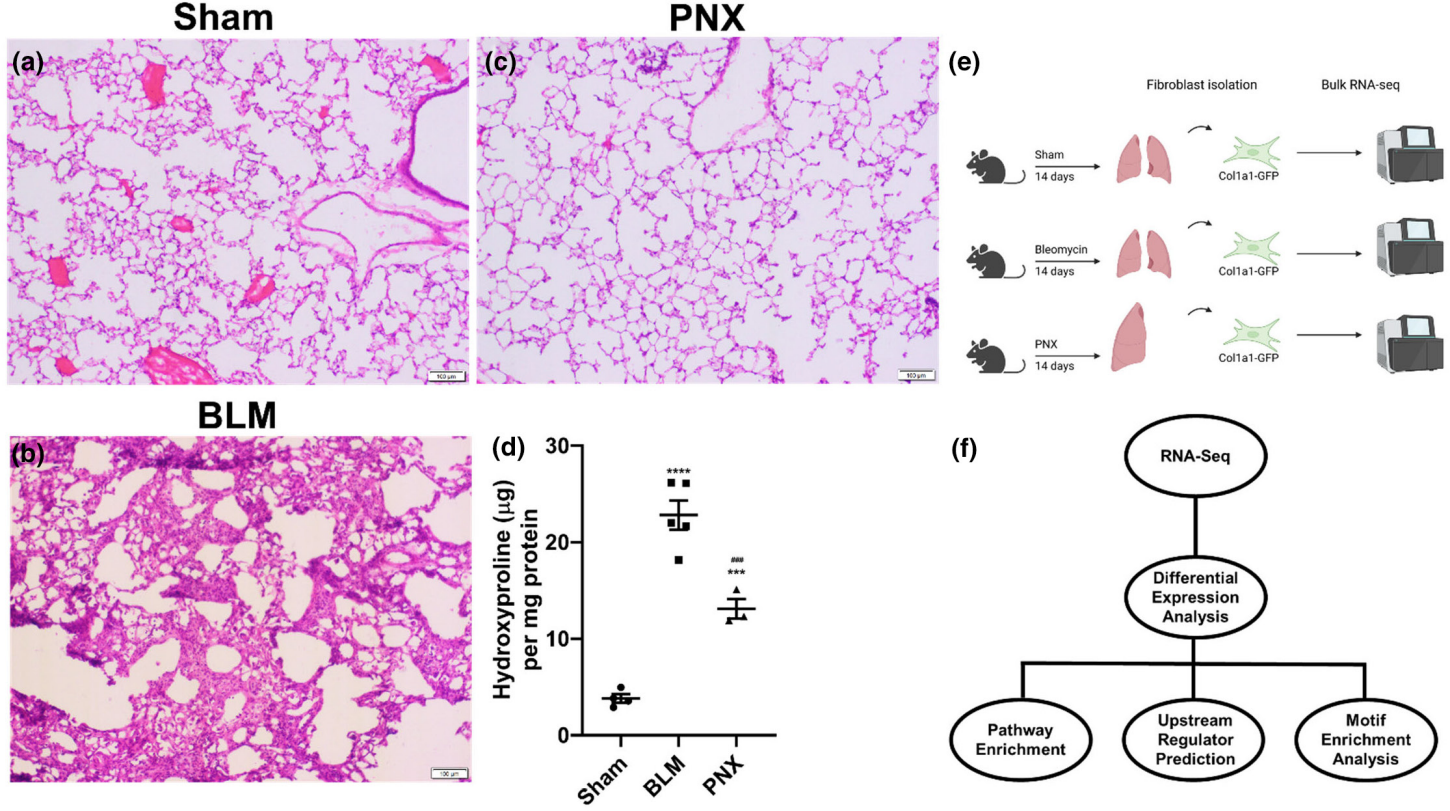
Experimental design and models of fibrosis and lung regeneration. (a–c): Hematoxylin and eosin staining of lung parenchyma following sham (a), bleomycin (BLM, b), and pneumonectomy (PNX, c) at 100× magnification. (d) Hydroxyproline assay showing increases in lung collagen content following BLM and PNX. (e) Schematic showing overall experimental workflow. (f) Diagram showing analysis plan for RNA‐sequencing. Statistical significance: ***p=0.0004 (comparing BLM with PNX), ****p<0.0001 (comparing BLM with Sham), ^###^ *p*=0.0008 (comparing PNX with Sham).

Fourteen days following either PNX or BLM challenge, lung tissue remodeling and collagen deposition were investigated by H&E staining and hydroxyproline assay, respectively. Expectedly, BLM caused marked thickening of the alveolar walls. In contrast, PNX did not cause any noticeable histologic changes (Figure 1a–c). Hydroxyproline assay indicated a robust increase in whole‐lung collagen content following either PNX or BLM, but the level of collagen deposition after PNX was significantly lower than after BLM (Figure 1d). As fibroblasts are the primary cell type responsible for collagen deposition (Tsukui et al., 2020), we then aimed to gain a deeper understanding of how they respond differently to BLM or PNX by performing bulk RNA‐sequencing and bioinformatic analyses on isolated lung fibroblasts (Figure 1e,f).

### 3.2 Overlap between fibroblast transcriptomic responses to BLM and PNX

**FIGURE 2:**
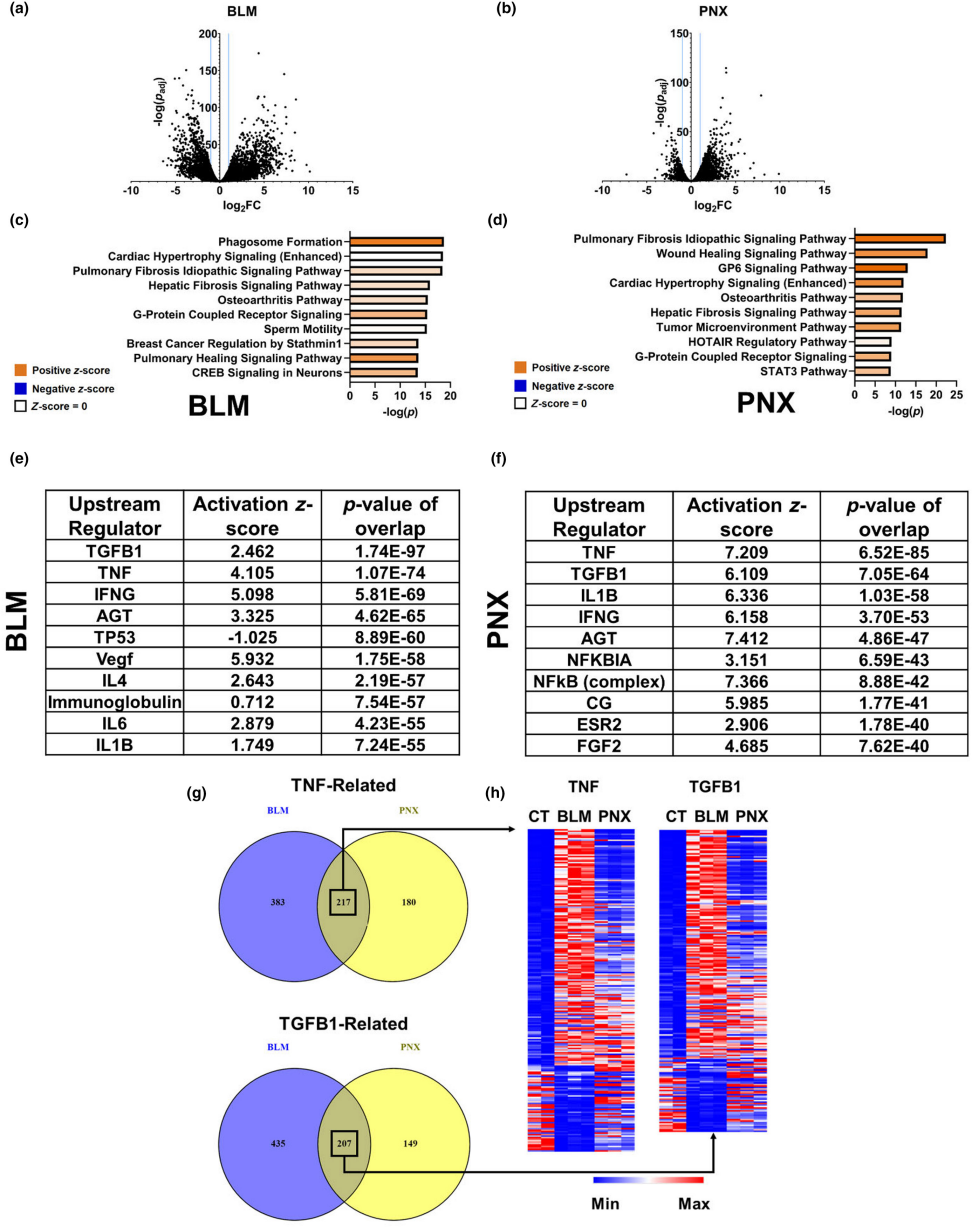
Transcriptomic response to BLM and PNX. (a, b): Volcano plots showing all protein‐coding genes from BLM (a) or PNX (b) fibroblasts with an adjusted *p*‐value less than 0.05 when compared to sham. Blue lines indicate an absolute log_2_ fold‐change of 1, used as a criterion for differential expression. (c, d): Bar graphs showing the top 10 enriched pathways by *p*‐value for BLM (c) and PNX (d) using Ingenuity Pathway Analysis (IPA). Positive z‐scores are associated with pathway activation based on gene fold‐changes. (e, f): Top 10 predicted upstream regulators using IPA for BLM (e) and PNX (f), sorted by *p*‐value. (g) Venn diagrams showing the number of genes used to predict TNF and TGFB1 as upstream regulators. The number in each region of the chart indicates the number of genes that were differentially expressed following the associated procedure. (h) Heatmaps showing relative expression for genes commonly differentially expressed following BLM and PNX associated with the TNF or TGFB1 upstream regulators. Heatmap colors are derived for scaled minimum and maximum for each row (gene).

RNA sequencing identified 1778 protein‐coding differentially expressed genes (DEGs) following PNX, which is much fewer than the 3668 DEGs following BLM in Col1a1‐GFP+ fibroblasts (Figure 2a,b, Data S1). To functionally determine the biological significance of these differential gene expression programs, we performed pathway enrichment analysis using Qiagen's Ingenuity Pathway Analysis (IPA) software. IPA runs a knowledge‐based prediction algorithm that accounts for the magnitude and direction of each DEG. Figure 2c,d highlight the top 10 most enriched biological processes among genes differentially regulated by either BLM or PNX, respectively. Notably, many processes were similar following either procedure, and were related to wound healing or fibrosis.

To identify differences between the responses to BLM and PNX, we also predicted upstream regulators based on DEGs using IPA. We identified considerable overlap between the most highly predicted regulators following BLM or PNX (Figure 2e,f). Many of the predicted upstream regulators for both procedures were growth factors such as TGF‐β1, or molecules related to immune signaling. Intrigued by this apparent similarity, we next asked which genes were linked to the top‐predicted upstream regulators. Figure 2g shows Venn diagrams displaying the number of DEGs unique to or shared by fibroblasts responding to either BLM or PNX for the top two predicted upstream regulators (*TNF* and *TGFB1*). Notably, despite the overlap in predicted upstream regulators, some distinct gene expression program subsets appeared to be engaged differentially in response to BLM and PNX. We next compared relative expressions of genes mapped to the *TNF* and *TGFB1* upstream regulators that were differentially regulated following both BLM and PNX. Among these sets of genes, we noted considerable differences in relative expression (Figure 2h). These results indicate that while upstream regulator prediction suggests a high degree of similarity between the two responses, there are considerable underlying differences related to the magnitude of gene expression changes in response to BLM and PNX. Data S2 contains a list of genes mapped to either the *TGFB1* or *TNF* upstream regulator that were differentially regulated following both procedures and their log_2_ fold‐change compared to sham mice.

### 3.3 Anti‐fibrotic pathways distinguish responses between PNX and BLM

**FIGURE 3:**
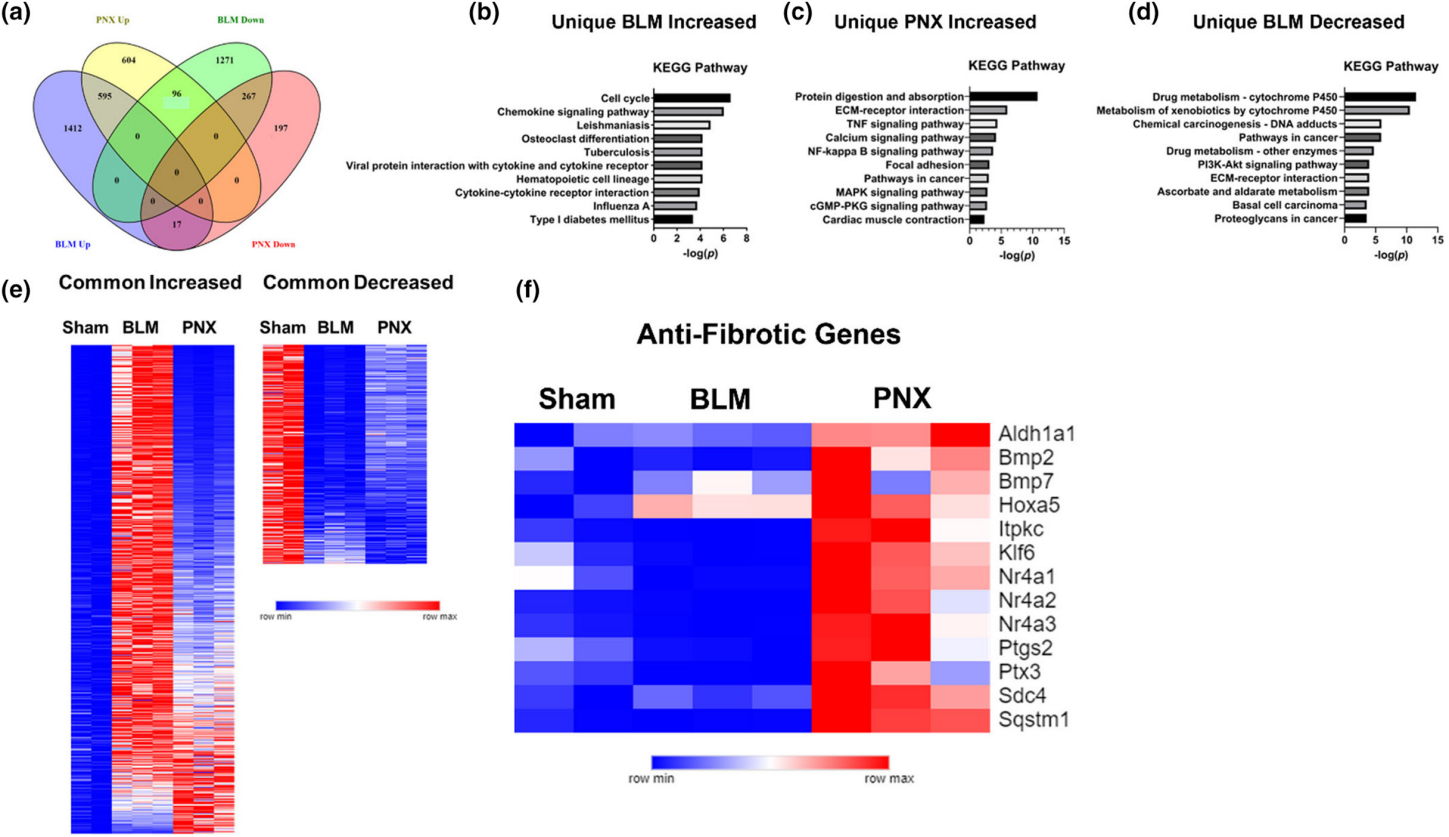
Transcriptome‐wide differences between the responses to BLM and PNX. (a): Venn diagram showing the number of common and unique genes that significantly increase or decrease in expression following BLM or PNX. (b–d): Bar graphs showing the top 10 enriched KEGG pathways enriched among the associated gene set. (e): Heatmaps showing relative expression of genes that significantly change in the same direction following BLM and PNX. (f): Heatmap showing expression of select previously described anti‐fibrotic genes increasing following PNX. Heatmap colors are derived for scaled minimum and maximum for each row (gene).

We next aimed to gain a deeper understanding of key differences between the responses to BLM and PNX. Figure 3a shows a Venn diagram of the total number of common and unique genes that significantly increase or decrease in expression following BLM or PNX. To characterize differences in the responses to BLM and PNX, we performed enrichment analysis for Kyoto Encyclopedia of Genes and Genomes (KEGG) pathways on genes that increased or decreased following only one procedure. The most significantly enriched KEGG pathways associated with genes only increased following BLM were mostly related to increased proliferation, chemokine production, and inflammation (Figure 3b). Top pathways for genes only increased by PNX were related to matrix digestion and absorption, TNF/NF‐κB signaling, and calcium signaling (Figure 3c). KEGG pathways enriched among genes that decreased only following BLM were linked closely to drug metabolism (Figure 3d). Only one KEGG pathway, related to antiviral signaling, was significantly enriched among genes that only decreased following PNX (not shown).

In line with above observations that shared DEGs may change at noticeably different magnitudes, we first examined genes which significantly change in the same direction following both BLM and PNX. Expression heatmaps (Figure 3e) show an exaggerated effect of BLM on expression of genes that also change in the same direction following PNX. Five hundred and ten of 595 genes (85.7%) that significantly increase during both processes increase more following BLM, whereas 200 of 267 (74.9%) common decreased genes decrease more following BLM. Furthermore, we identified several genes previously documented to moderate pro‐fibrotic responses uniquely upregulated after PNX but not BLM (Figure 3f), including *Ptgs2*, responsible for synthesis of the antifibrotic prostaglandin E2 (Bauman et al., 2010; Huang et al., 2007; Keerthisingam et al., 2001; Lama et al., 2002), Bone Morphogenetic Protein (BMP) ligands (*Bmp2*, *Bmp7*) (De Langhe et al., 2015; Guan et al., 2022; Pegorier et al., 2010; Tan et al., 2019), the Nuclear Receptor 4A (NR4A) Family (*Nr4a1*, *Nr4a2*, *Nr4a3*), and the aldehyde dehydrogenase (ALDH) gene superfamily (*Aldh1a1*) (Ma et al., 2018; Takahashi et al., 2021; Wang et al., 2023). These previously described anti‐fibrotic transcripts elevated after PNX but not BLM might contribute to the lower magnitude of fibrotic responses in PNX compared with BLM (Figure 3e).

### 3.4 ITPKC is differentially regulated after BLM and PNX

**FIGURE 4:**
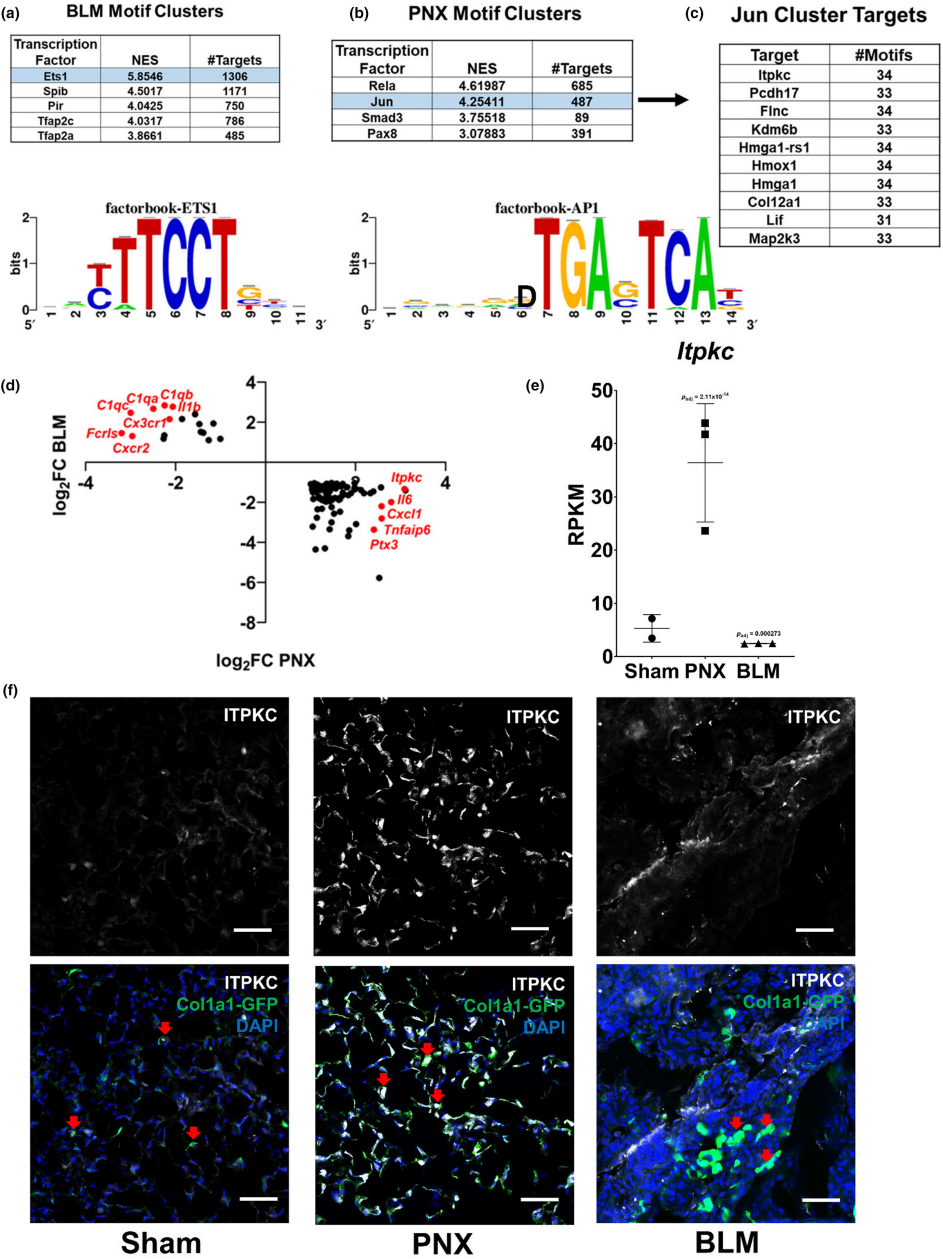
Identification of ITPKC. (a, b): Top 5 most enriched motif clusters following BLM (a) and PNX (b) and the associated position weight matrix of the Ets1 and Jun motifs. A higher normalized enrichment score (NES) indicates stronger enrichment. (c): Table showing the top 10 predicted targets among genes up‐regulated by PNX associated with the Jun cluster; and the number of motifs in that cluster associated with the target. (d): Plot showing the fold‐change of genes differentially expressed in opposite directions following BLM or PNX. *Itpkc* and innate immune genes are highlighted in red. (e): *Itpkc* expression expressed as reads per kilobase of transcript per million reads mapped (RPKM) across conditions with the indicated adjusted *p*‐value as determined by the DESeq2 algorithm. (f): Representative immunofluorescence images of IPTKC expression in the Col1a1‐GFP mice (Red arrows indicated representative Col1a1‐GFP cells) at D14 following Sham, BLM and PNX treatment. Scale bar = 50 μm.

To further understand the responses to BLM and PNX, we next employed motif enrichment analysis using the software iRegulon (Janky et al., 2014). This software takes a list of genes as input, and outputs a list of transcription factor motifs enriched according to the input gene set. Our input lists contained only genes up‐regulated following the respective procedure compared to sham mice. Accordingly, the most enriched motifs are those that would be predicted to be the most accessible during the associated response. Figure 4a (Data S3) shows the top five most enriched motif clusters associated with genes up‐regulated by BLM. Each cluster contains similar transcription factor binding motifs and is marked by the most enriched motif for that cluster. The response to BLM is strongly characterized by motifs associated with ETS1 and SPIB (Transcription factor Spi‐B) family members. Inputting genes up‐regulated by PNX revealed strong enrichment for motifs similar to RELA and JUN (Figure 4b, Data S3). RELA and JUN are members of the NF‐κB and AP‐1 families, respectively. Interestingly, the top‐ranked target associated with the JUN motif cluster was *Itpkc*, a gene whose role in lung fibroblasts has yet to be described (Figure 4c).

Among genes regulated in different directions by BLM and PNX, *Itpkc* was one of the most highly up‐regulated genes following PNX (Figure 4d). *Itpkc* was not highly expressed under sham conditions and was slightly repressed following BLM (Figure 4e). Immunostaining of ITPKC is much higher in the Col1a1‐GFP fibroblast (arrowed) from PNX than those cells (arrowed) from either BLM or Sham (Figure 4f). Interestingly, many of the genes regulated the most differently by BLM and PNX were involved in immune and inflammatory signaling (Figure 4d). An *ITPKC* loss‐of‐function mutation has previously been found to be associated with the vascular autoimmune syndrome Kawasaki disease (Onouchi et al., 2008), indicating a possible link to inflammatory signaling and prompting us to investigate this gene further.

### 3.5 ITPKC moderates pro‐fibrotic responses

**FIGURE 5:**
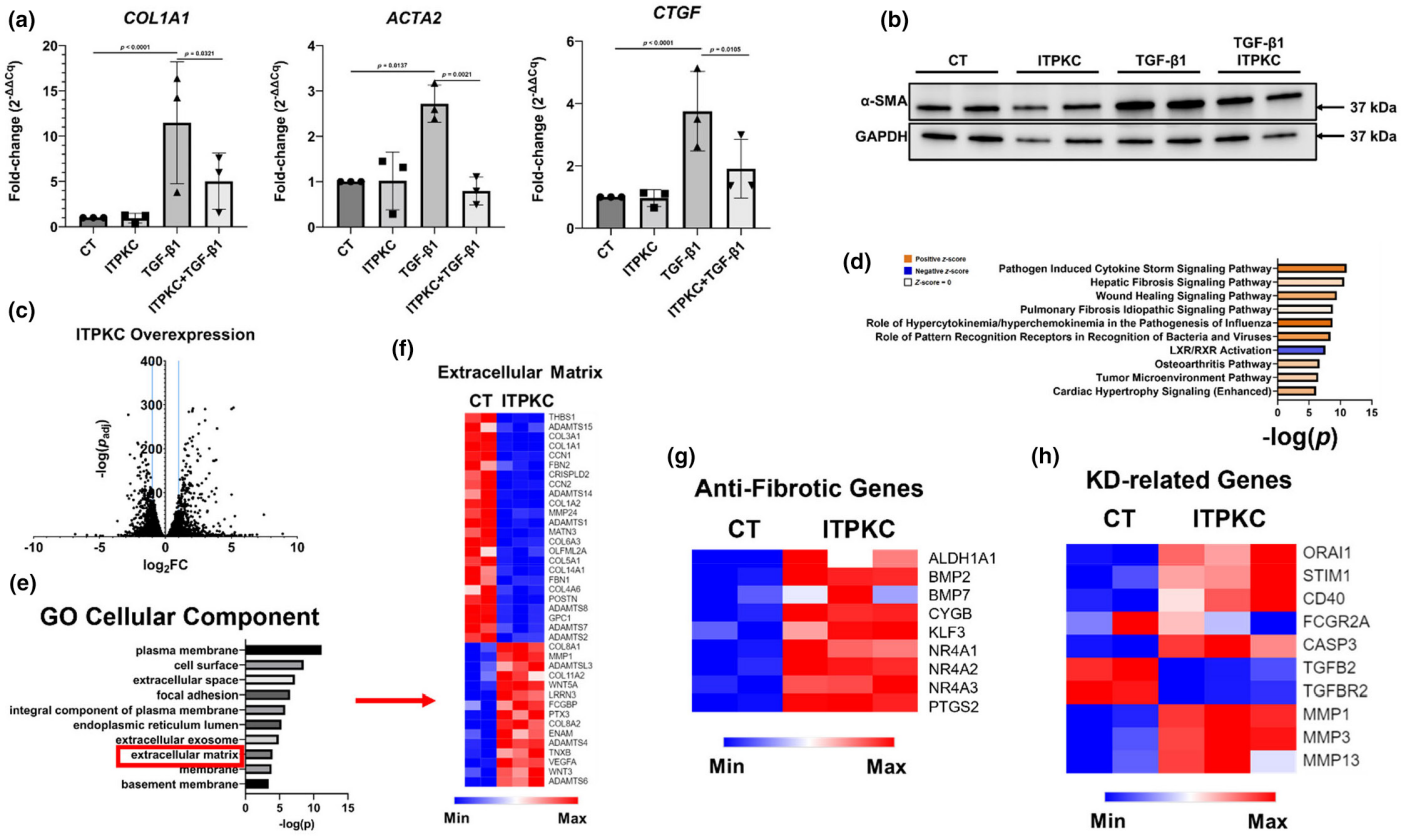
Anti‐fibrotic effect of ITPKC (a): Quantitative real‐time PCR for fibrogenic markers *COL1A1*, *ACTA2*, and *CTGF* in primary normal human lung fibroblasts in the presence or absence of ITPKC overexpression and/or TGF‐β1. Values are expressed as fold‐change (2−^ΔΔC^_q_). Each data point represents an independent experiment, with each experiment containing three technical replicates. Statistical significance was determined using one‐way ANOVA with Fisher's least significant difference as a post‐hoc test. Statistical analyses were conducted on ΔC_q_ values compared to *GAPDH*. (b): Western blot showing α‐smooth muscle Actin (SMA) expression in primary normal human lung fibroblasts in the presence or absence of ITPKC overexpression with or without TGF‐β1. (c): Volcano plot showing all protein‐coding genes from ITPKC‐overexpressing NHLFs with an adjusted *p*‐value less than 0.05 compared to control. Blue lines indicate an absolute log_2_ fold‐change of 1, used as a criterion for differential expression. (d) Bar graph showing the top 10 enriched pathways by *p*‐value associated with ITPKC overexpression using IPA. Positive *z*‐scores are associated with pathway activation based on differential gene expression. (e): Bar graph showing the top 10 cellular compartments associated with genes differentially expressed following ITPKC overexpression, sorted by adjusted *p*‐value. (f): Heatmap showing relative expression of genes associated with the extracellular matrix compartment for control and ITPKC‐overexpressing NHLFs. (g): Heatmap showing expression of select anti‐fibrotic genes following ITPKC overexpression. (h): Heatmap showing expression of genes associated with Kawasaki disease. Heatmap colors are derived for scaled minimum and maximum for each row (gene).

To investigate functional roles of ITPKC, we transiently transfected primary normal human lung fibroblasts (NHLFs) with recombinant human *ITPKC* (Figure S1) and used exogenous TGF‐β1 to model fibrotic changes. ITPKC overexpression reduced the effects of TGF‐β1 on genes encoding collagen‐1α1 (*COL1A1*), α‐smooth muscle actin (*ACTA2*), and connective tissue growth factor (*CTGF*) (Figure 5a). ITPKC overexpression also reduced TGF‐β1‐mediated α‐SMA up‐regulation at the protein level (Figure 5b).

Encouraged by these findings, we aimed to determine the effects of ITPKC overexpression in more detail using RNA‐seq. ITPKC overexpression in NHLFs resulted in up‐regulation of 736 and down‐regulation of 619 protein‐coding genes (Figure 5c and Data S4). IPA revealed enrichment for pathways mostly related to cytokine signaling, wound healing, and fibrosis (Figure 5d). DEGs resulting from ITPKC overexpression were mostly linked to the plasma membrane and secreted cellular components, including the extracellular matrix (ECM) (Figure 5e). Figure 5f shows down‐regulation of several collagen genes, and the elastic fiber components fibulin‐1 and ‐2. Up‐regulated genes include collagen‐degrading matrix metalloproteinase 1 and vascular endothelial growth factor A, which has been shown to be protective against BLM‐induced lung fibrosis (Murray et al., 2017). Similarly, IPTKC overexpression significantly increased several anti‐fibrotic genes that were also increased following PNX including *PTGS2*, *BMP2*, *BMP7*, *NR4A1*, *NR4A2*, *NR4A3*, and ALDH family member *ALDH1A2* (Figure 5g). Finally, taking into consideration the causal associated between *ITPKC* polymophisms and Kawasaki disease (Onouchi et al., 2008), we have noted that overexpression of ITPKC in lung fibroblasts results in enhanced T cell activation (*ORAI1*, *STIM1*), which is associated with Kawasaki disease (Murray et al., 2017). More importantly, it leads to enhanced apoptosis (*CASP3*), decreased transforming growth factor‐beta signaling (*TGFB2*, *TGFBR2*) and increased ECM degradation (*MMP1*, *MMP3*, *MMP13*) (Murray et al., 2017), all of which are critical for fibrosis resolution (Figure 5h). These observations suggest that ITPKC plays an anti‐fibrotic role in fibroblasts.

### 3.6 ER calcium transport is required for the fibrogenic effects of TGF‐β1

ITPKC is an enzyme that catalyzes the conversion of IP3 to IP4. IP3 binds to and activates IP3 receptors (IP3Rs), which are calcium channels localized to the endoplasmic reticulum (ER). Upon activation, IP3Rs increase intracellular Ca^2+^ levels by allowing Ca^2+^ to leave the ER. To confirm the dependency of fibroblast activation by TGF‐β1 on IP3‐mediated Ca^2+^ release, we treated NHLFs with TGF‐β1 in the presence or absence of the IP3R inhibitor 2‐Aminoethoxydiphenyl borate (2‐APB). As predicted, 2‐APB reduced the effects of TGF‐β1 on *COL1A1*, *ACTA2*, *CTGF*, and fibronectin 1 (*FN1*) as determined by qRT‐PCR (Figure S2a), similar to our observations with overexpression of ITPKC. Thapsigargin, which raises intracellular Ca^2+^ levels by preventing ER calcium uptake by inhibiting the sarco/endoplasmic reticulum ATPase (SERCA), had a similar effect to 2‐APB (Figure S2b). This is in agreement with reports that Ca^2+^ oscillations are an important signal in mediating the pro‐fibrotic effects of TGF‐β1 (Mukherjee et al., 2012), because our pharmacological interventions shift intracellular Ca^2+^ ion distribution to either the ER (2‐APB) or the cytosol (Thapsigargin).

## 4 DISCUSSION

Here, we have conducted RNA‐sequencing on lung fibroblasts freshly isolated from mice after PNX or BLM. Both PNX and BLM caused an increase in collagen in the lungs and activated similar gene transcriptional programs, though with more muted responses in fibroblasts post‐PNX. We identified *Itpkc* as a gene uniquely upregulated following PNX; and demonstrated that overexpression of ITPKC in human lung fibroblasts attenuates pro‐fibrotic responses to TGF‐β1 and enhances expression of several other transcripts previously shown to moderate pro‐fibrotic responses in vivo.

PNX provides a useful platform to study lung regeneration in an adult animal model, particularly as a contrast with BLM, which produces dysregulated and fibrotic repair over the same time courses. At the 14‐day time point, many of the genes regulated the most differently in PNX versus BLM were related to anti‐fibrotic pathways (Figure 3f), which prevent excessive fibrotic responses and matrix deposition while promoting homeostatic or regenerative lung repair. *PTGS2* encodes cyclooxygenase‐2, which is involved in prostaglandin‐E2 (PGE2) synthesis. PGE2 exhibits strong anti‐fibrotic effects in the lung (Bauman et al., 2010; Huang et al., 2007; Keerthisingam et al., 2001; Lama et al., 2002). The BMP family has also been well‐described to exhibit anti‐fibrotic effects (De Langhe et al., 2015; Guan et al., 2022; Pegorier et al., 2010; Tan et al., 2019). The anti‐fibrotic effects of the nuclear receptor family and aldehyde dehydrogenase (*ALDH*) gene superfamily have been shown in our previous work (Tan et al., 2021) and by others (Ma et al., 2018; Takahashi et al., 2021; Wang et al., 2023). Pentraxin 3 (*Ptx3*) has been shown to protect against BLM‐induced lung fibrosis in mice as well (Maccarinelli et al., 2021). Notably, overexpression of ITPKC increased expression of many of these anti‐fibrotic transcripts, suggesting its broad importance for moderating fibroblast responses that could otherwise result in fibrotic outcomes.

ITPKC catalyzes the conversion of IP3 to IP4, which effectively prevents activation of IP3Rs and therefore reduces Ca^2+^ release from the ER (Dewaste et al., 2003). Considering the function of ITPKC, we confirmed that the IP3R inhibitor 2‐APB could also prevent the fibrogenic effects of TGF‐β1 (Figure S2a). Others have shown that intracellular Ca^2+^ oscillations, rather than static concentration, are important signals for mediating the fibrogenic effects of TGF‐β1 in human lung fibroblasts (Mukherjee et al., 2012). The authors of this study attributed this effect to ryanodine receptors, another family of ER‐localized Ca^2+^ channels (Mukherjee et al., 2012). However, our mouse RNA‐seq data suggests that these genes are very lowly expressed in lung fibroblasts under the conditions we analyzed (Data S1).

Supporting a potentially therapeutically relevant role for intracellular Ca^2+^ in fibrotic activation, another study has shown adeno‐associated virus‐mediated delivery of the *SERCA2a* gene to alleviate bleomycin‐induced pulmonary fibrosis in mice, as well as TGF‐β1‐mediated fibroblast‐myofibroblast transition in NHLFs (Bisserier et al., 2020). *SERCA2a* encodes one paralog of the sarco/endoplasmic reticulum Ca^2+^‐ATPase, which actively pumps Ca^2+^ ions from the cytosol into the ER. SERCA overexpression would be expected to have similar effects to ITPKC overexpression, because ITPKC limits Ca2+ release from the ER through IP3 receptors. While we acknowledge that TGF‐β does not completely mimic the biology of fibrosis, these findings may also indicate that targeting the IP3 receptors could offer benefit in pulmonary fibrosis and highlights the potential translational relevance of our findings.

**FIGURE 6:**
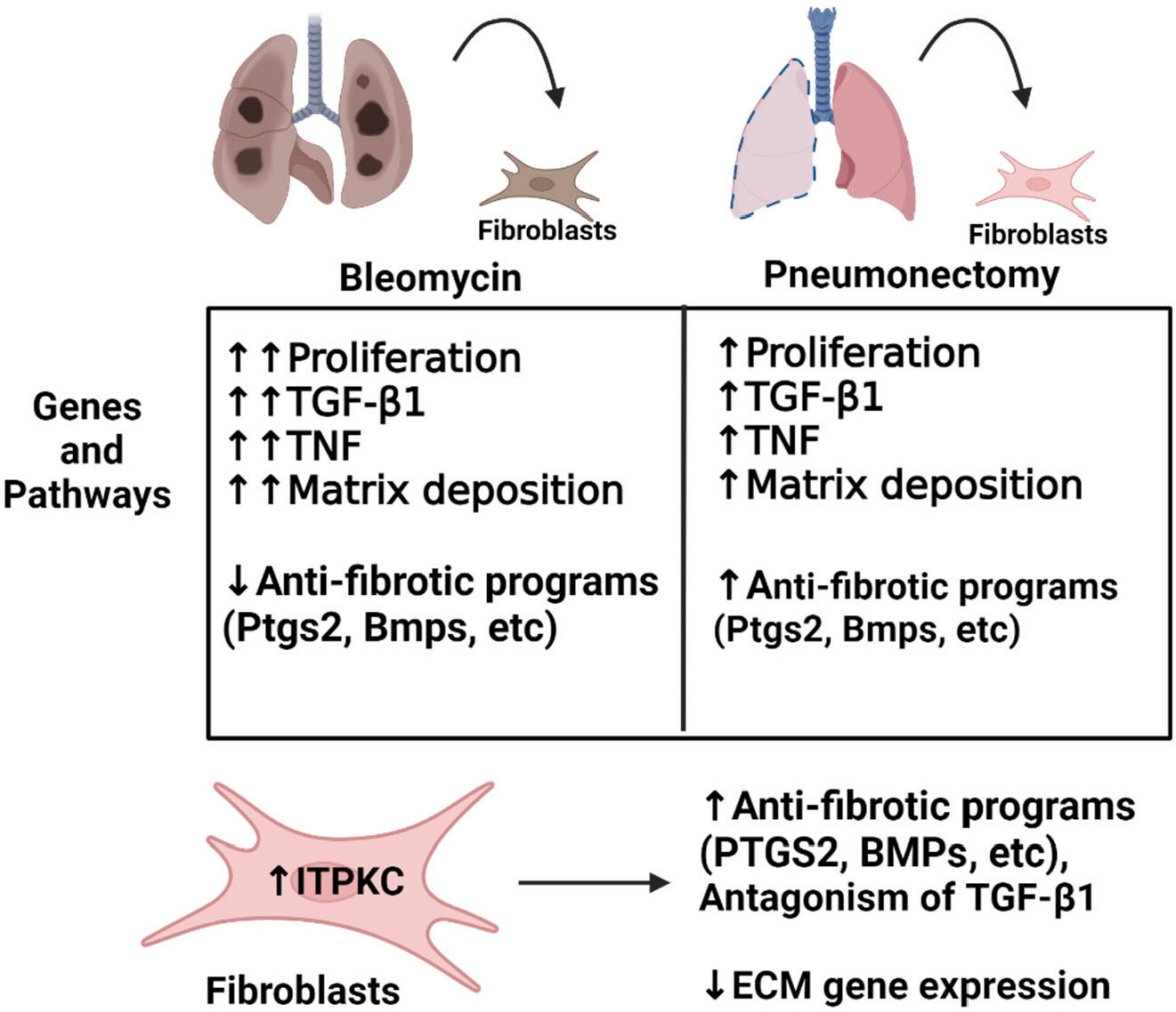
Diagram summarizing key findings of this study. We used RNA‐sequencing to make comparisons in lung fibroblasts isolated from mice undergoing pneumonectomy or bleomycin exposure. The table highlights that pneumonectomy causes a lesser fibrotic response compared with BLM, indicating that repressed anti‐fibrotic programs in bleomycin induces fibrotic lung repair and upregulated anti‐fibrotic programs in pneumonectomy promotes regenerative lung repair. ITPKC, related to calcium signaling, was a key difference we identified and validated in vitro. Overexpression of ITPKC in human lung fibroblasts attenuates the fibrogenic effects of TGF‐β1 and induces anti‐fibrotic programs similar to that seen with pneumonectomy.

Taken together, our results highlight surprisingly similar, though more muted responses of pulmonary fibroblasts to PNX compared to BLM; and identified ITPKC and multiple known anti‐fibrotic transcripts as potential moderators of fibroblast responses to PNX Figure 6. Our comparative RNA‐seq strategy thus helped to identify a novel candidate pathway that moderates pro‐fibrotic responses, potentially pointing toward therapeutic approaches that may help to attenuate fibroblast responses in fibrotic lung disease. Future delineation of the cues and combinatorial signals that subtly shift fibroblast responses toward regenerative versus fibrotic outcomes will lead to a better understanding of fibroblast biology in these contexts, with our RNA‐seq results serving as a valuable starting point for such investigations.

## AUTHOR CONTRIBUTIONS

J.H.W. designed and performed cell culture experiments, analyzed and interpreted data from cell culture and bioinformatic experiments, wrote the manuscript, and prepared figures. S.R.D., J.A.M., performed animal experiments. K.M.C. performed hydroxyproline assays. D.J.T. designed experiments and revised the manuscript. Q.T. designed and performed animal experiments, analyzed data, wrote and revised the manuscript for important intellectual content. All authors read and approved the manuscript.

## FUNDING INFORMATION

The authors acknowledge funding for JHW from the T32 grant HL105355, an NIH R01 grant HL092961 awarded to DJT and an NIH R01 grant HL153026 awarded to QT.

## CONFLICT OF INTEREST STATEMENT

The authors declare that they have no competing interests.

## ETHICS STATEMENT

All mouse experiments were performed under a protocol approved by the Mayo Clinic Institutional Animal Care and Use Committee.

## Supporting information


**Figure S1.** Quantitative real‐time PCR for ITPKC in primary normal human lung fibroblasts transiently overexpressing ITPKC or control. Data were generated from 3 biological replicates, with each having 3 technical replicates. Statistical significance was determined using Student’s two‐tailed *t*‐test on ΔCt values compared to GAPDH.
**Figure S2.** Quantitative real‐time PCR for fibrogenic markers COL1A1, ACTA2, CTGF, and FN1 in primary normal human lung fibroblasts in the presence or absence of TGF‐β1 with or without (a) 50 μM 2‐Aminoethoxydiphenyl borate (2‐APB) or 5 μM thapsigargin (b). Values are expressed as fold‐change (2^−^ΔΔCt). Data were generated from 3 technical replicates. Statistical significance was determined using one‐way ANOVA with Fisher’s least significant difference as a posthoc test. Statistical analyses were conducted on ΔCt values compared to GAPDH.

**Data S1.** RNA‐Sequencing of isolated mouse lung fibroblasts following bleomycin administration or pneumonectomy.

**Data S2.** Table of genes differentially express following both BLM and PNX that were mapped to the upstream regulators TGFB1 and TNF showing their log2 fold‐change compared to sham mice for either procedure.

**Data S3.** Motif enrichment prediction for genes up‐regulated in mouse lung fibroblasts following bleomycin or pneumonectomy.

**Data S4.** RNA‐Sequencing of cultured normal human lung fibroblasts overexpressing ITPKC.

## Data Availability

Data will be made available upon reasonable request.
