# Supplementary material for: RNA‐sequencing reveals differential fibroblast responses to bleomycin and pneumonectomy
Source: Physiol Rep. 2024 Jul 11;12(13):e16148. doi: 10.14814/phy2.16148 (PMC11239319; doi:10.14814/phy2.16148)
Supplement: Supplementary file 1 — Figure S1. Quantitative real‐time PCR for ITPKC in primary normal human lung fibroblasts transiently overexpressing ITPKC or control. Data were generated from 3 biological replicates, with each having 3 technical replicates. Statistical significance was determined using Student’s two‐tailed t‐test on ΔCt values compared to GAPDH. Figure S2. Quantitative real‐time PCR for fibrogenic markers COL1A1, ACTA2, CTGF, and FN1 in primary normal human lung fibroblasts in the presence or absence of TGF‐β1 with or without (a) 50 μM 2‐Aminoethoxydiphenyl borate (2‐APB) or 5 μM thapsigargin (b). Values are expressed as fold‐change (2−ΔΔCt). Data were generated from 3 technical replicates. Statistical significance was determined using one‐way ANOVA with Fisher’s least significant difference as a posthoc test. Statistical analyses were conducted on ΔCt values compared to GAPDH. [file PHY2-12-e16148-s002.docx]

**SUPPLEMENTAL DATA**

**
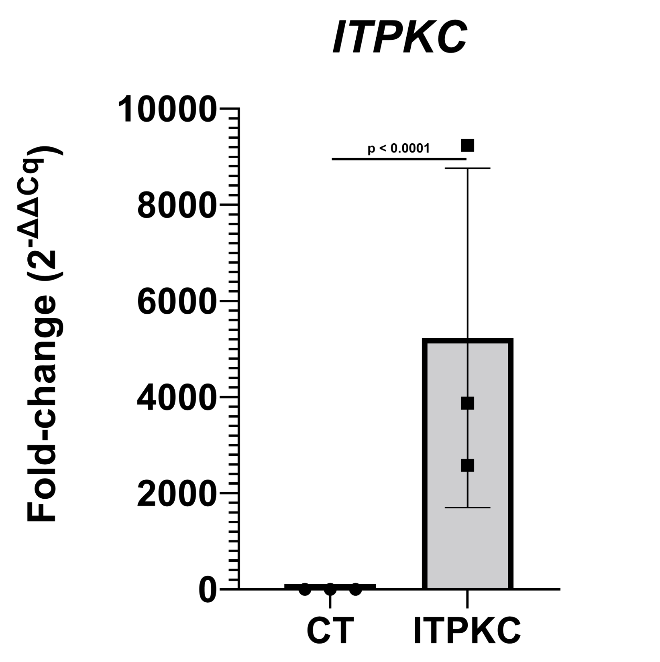
**

**Figure S1**

Quantitative real-time PCR for *ITPKC* in primary normal human lung fibroblasts transiently overexpressing ITPKC or control. Data were generated from 3 biological replicates, with each having 3 technical replicates. Statistical significance was determined using Student’s two-tailed t-test on ΔC_t_ values compared to *GAPDH*.


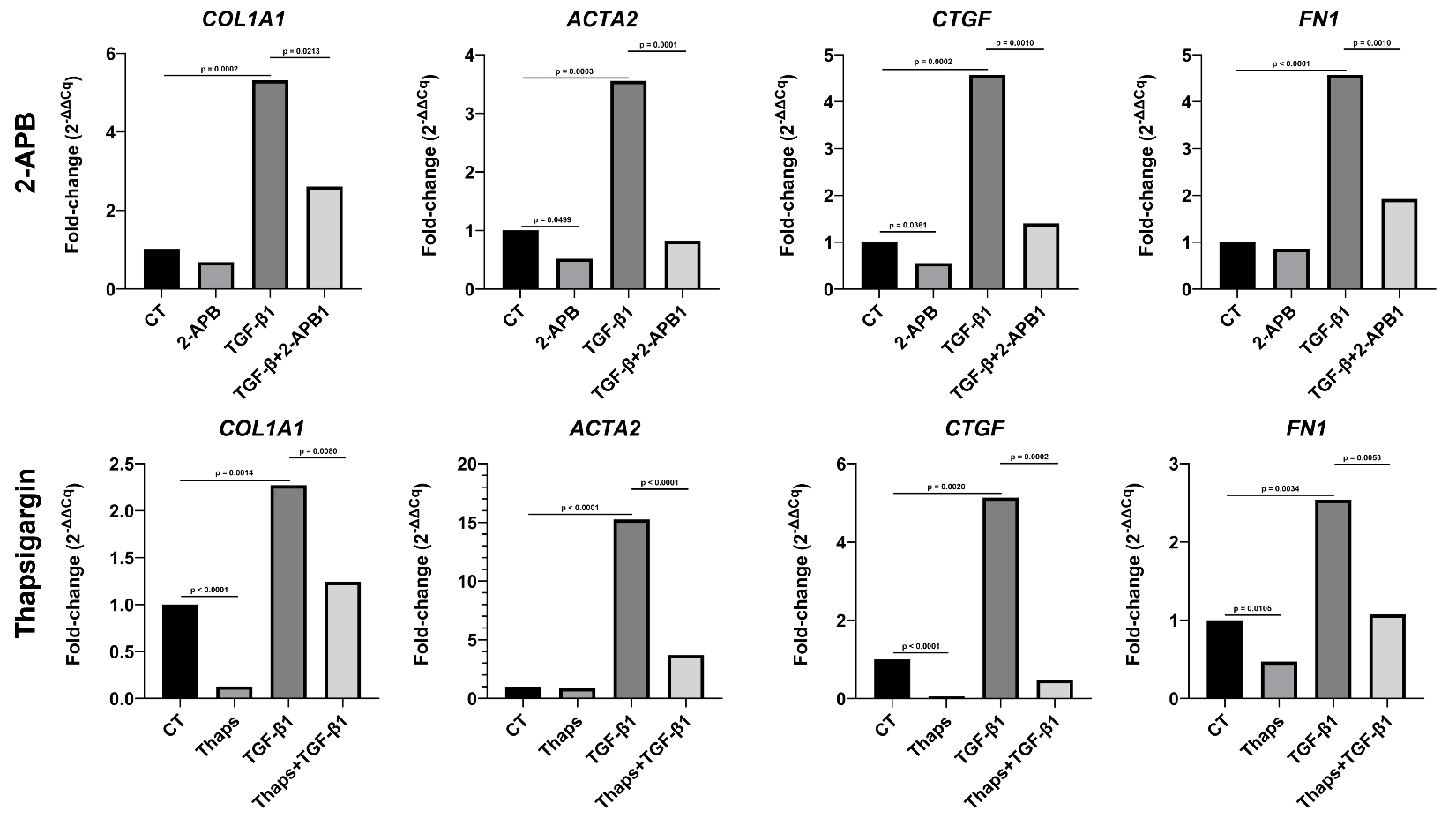


**Figure S2**

Quantitative real-time PCR for fibrogenic markers *COL1A1*, *ACTA2*, *CTGF*, and *FN1* in primary normal human lung fibroblasts in the presence or absence of TGF-β1 with or without **(A)** 50µM 2-Aminoethoxydiphenyl borate (2-APB) or 5µM thapsigargin **(B)**. Values are expressed as fold-change (2^-ΔΔC^_t_). Data were generated from 3 technical replicates. Statistical significance was determined using one-way ANOVA with Fisher’s least significant difference as a posthoc test. Statistical analyses were conducted on ΔC_t_ values compared to *GAPDH*.
